# Astrocyte Antioxidant Systems

**DOI:** 10.3390/antiox7090112

**Published:** 2018-08-27

**Authors:** Gethin J. McBean

**Affiliations:** School of Biomolecular and Biomedical Science, Conway Institute, University College Dublin, Dublin, Ireland; gethin.mcbean@ucd.ie

One of the most intriguing aspects of cerebral homeostasis concerns the relationship between astrocytes and neurons in ensuring neuronal well-being and stability. This is particularly true in the context of antioxidant defence. Astrocytes play a significant role in maintaining the army of antioxidants upon which neurons rely for their protection from oxidative stress. In the 20 years or so since Dringen and colleagues [1] first provided an insight into how astrocytes first synthesise and then export glutathione to neurons, much information has emerged on how astrocyte antioxidant mechanisms respond to conditions of oxidative stress, inflammation or infection. With our increasing knowledge of astrocyte antioxidant systems comes the realisation that these cells have the potential for delivering new therapies that have the power to enhance neuronal survival. 

This Special Issue provides review articles and original papers that describe new insights into mechanisms of astrocyte-mediated antioxidant defence. In their paper, Chowdhury et al. [2] investigate the antioxidant capacity of the pro-inflammatory cytokine, IL-1β. Having already established that IL-1β acts through up-regulation of glutathione production to protect astrocytes against oxidant damage [3], they now show that IL-1β also protects neurons from oxidative stress. Significantly, however, the response of IL-1β to oxidative stress is mediated via enhancing the synthesis and release of glutathione from astrocytes. This is a prime illustration of the neuroprotective capacity of astrocytes that operates via regulating the supply of glutathione to neurones. 

In the second article of this issue, Messina et al. [4] examine the role of small GTP-binding proteins of the p21^Ras^ family that are highly expressed in astrocytes. Two members of this family, H-Ras and K-Ras respond to changes in reactive oxygen species and regulate cellular redox state through induction of antioxidant genes that include Mn-superoxide dismutase and NADPH oxidase. They report that acute oxidative stimulation of primary astrocytes causes upregulation of K- and H-Ras via processes that target both the transcriptional and translational pathways. These observations have led the authors to propose that K- and H-Ras operate as sensors to changes in ROS production that underlie an astrocyte’s ability to increase its antioxidant capacity in response to oxidative stress. 

The review article by Liddell [5] provides a timely up-date on the workings of the transcription factor, Nrf2, which is frequently described as a “master regulator” of antioxidant genes. Following a detailed description of the mechanism of activation and regulation of Nrf2, the author then turns to examining the role of Nrf2 in specific examples of neurodegenerative disease, including Parkinson’s, Alzheimer’s and Huntington’s diseases, amyotrophic lateral sclerosis and multiple sclerosis. Whilst much evidence suggests that Nrf2 is activated in these diseases, it is less clear whether changes to Nrf2-mediated signalling are confined to astrocytes or to neurons. The emerging picture is that each disorder may be characterised by an individual pattern of cell-type selective changes in Nrf2 signalling. An enduring curiosity, however, is that, regardless of the disease, endogenous Nrf2 responses fail in their function of preventing oxidative stress and maintaining normal neuronal activity. 

Finally, McBean [6] looks at thiol redox balance in astrocytes, focussing particularly on cysteine/cystine and reduced/oxidised glutathione redox couples. She reviews the current status of our understanding of the biochemical pathways that contribute to glutathione synthesis in astrocytes, and their mechanism of regulation. Several questions remain, not least the relationship between the supply of cysteine for glutathione production, as opposed to its role as a precursor for other bioactive molecules, for example, hydrogen sulfide. As is highlighted in this and the other contributions to this issue, the role of astrocytes in providing antioxidant protection for neurons is supremely important and needs further investigation so that the potential of these cells as a therapeutic target in promoting antioxidant defence can be fully realised. 
